# OCCUPATIONAL ALLERGIC CONTACT DERMATITIS AMONG CONSTRUCTION WORKERS IN INDIA

**DOI:** 10.4103/0019-5154.53192

**Published:** 2009

**Authors:** Nilendu Sarma

**Affiliations:** *From Department of Dermatology, Venereology and Leprology, NRS Medical College and Hospital, 138 AJC Bose Road, Kolkata - 700014, India*

**Keywords:** *Cement*, *chromate*, *construction workers*, *India*, *occupational contact dermatitis*

## Abstract

**Background:**

Allergic contact dermatitis is one of the important occupational hazards in construction workers and it often leads to poor quality of life of the workers with substantial financial loss. However, this is often a neglected entity. There are no past studies on the construction workers in Indian subcontinent.

**Objective:**

This pilot study has been done to assess the allergological profile among the workers engaged in construction of roads and bridges.

**Materials and Methods:**

The study was conducted among the workers working on construction of a bridge, flyover, and roads in West Bengal, India. Sixteen workers were selected on clinical suspicion. Ten were selected randomly and patch tested with Indian standard battery of patch test allergens. Analysis of reactions and relevance of positive test was assessed as per standard guidelines.

**Results:**

All the workers were men. Average age of workers was 24.8 years (range, 19-34 years). Dermatitis affected exposed parts in 93.75% and covered areas in 62.5%. Total positive test was 24 and relevant 11. Most common allergens were chromate (relevant allergy/RA: in 60% of patch tested workers), epoxy resin (RA: 30%), cobalt (RA: 20%), nickel (RA: 20%), thiuram mixture (RA: 10%) and black rubber mix (RA: 10%). Two cases (20%) had irritant contact dermatitis.

**Conclusion:**

The result indicated that chromate is the most frequent allergen among construction workers in this part of India. High frequency of involvement of the covered areas as well as the exposed areas highlighted the fact that the allergens had access to most body parts of the workers.

## Introduction

Occupational contact dermatosis (OCD) is the most significant and frequent dermatoses among all occupational skin diseases (OSDs). They are almost equivalent.[1] OCD is a significant occupational hazard in some jobs, especially in the construction industry. Cement is one of the most important cause of occupational disease in construction workers. Reported prevalence of allergic contact dermatitis (ACD) to chromate among this population usually is more than 10%.[2] The prevalence among symptomatic construction workers who were patch tested is more than 45%.[3] Such high prevalence is in sharp contrast with the rate of around 1% among the general population.[4]

Apart from chromate, epoxy resin,[3] cobalt, nickel,[5] and rubber chemicals[6] are also important allergens in construction industry.

Diagnosis and management of occupational skin disease (OSD) is often inadequate.[7] It is even more poorly addressed in resource-limited countries, including India. There are no studies to date on the construction workers in Indian subcontinent. This pilot study has been done to assess the allergological profile among the symptomatic workers engaged in construction of roads and bridges.

## Materials and Methods

The study was conducted in 2004 among workers engaged in construction of a bridge across the river Ganga, flyover across nearby railway tracks and link roads in Hooghly and Howrah districts of West Bengal. The study was initiated with the request from the private company under which the construction work was going on. Many of the workers developed some itchy rashes after commencement of the work.

A visit was conducted to the site camp. A detailed enquiry was made on the exact nature of work, time since the workers were on the job and the time of onset of the symptoms. They were examined to note the symptoms, distribution of disease, clinical pattern of the lesions, any other skin disease and past history of similar or other past skin illness.

All patients with itchy dermatitic conditions that aggravated or started for the first time after joining this work were included. The diseases excluded were infective diseases like scabies, fungal infections, bacterial infections and all noneczematous dermatoses like psoriasis and lichen planus, even if associated with itching. A clinical diagnosis of common itchy skin disease like atopic dermatitis were not regarded as a basis for exclusion if there was a clearly observable aggravation noted by patient after joining the job. Distribution of the condition was not a limiting factor and lesions over all areas, including exposed as well as covered areas, were included. However, diseases solely affecting the groins and genitals were excluded.

Among 28 workers examined, 12 were excluded (infective disease in 9 and persistent dermatitis without any obvious aggravation in 3) and 16 workers were selected. All had eczematous skin conditions that either started for the first time or aggravated significantly after joining the job.

Routine blood tests including total and differential count, hemoglobin, ESR, urea, creatinine and routine examination of urine were advised in most of the study participants.

All patients were patch tested with Indian Standard Battery (marketed by Systopic laboratories limited) of patch test allergens. Allergens were put into aluminium chambers and occluded for 48 hours. Test site was examined at 30 minutes after removal of patches and then at 96 hours and again on 7^th^ day.

Analysis of reactions and relevance of positive test was assessed as per standard guidelines.

## Results

At the time of this study, the project was few months old. All workers were on job for an average of seven months. All workers were men and their average age was 24.8 years (age range, 19-34 years) [Table 1].

**Table 1:** Clinico-allergological details of each patients

Age (years)	Distribution	AD	Atopy	Number of positive tests	Relevant allergens
19	Exp>Cov	N	n	7	Chromate, nickel
23	Exp	N	Y	5	Chromate, black rubber mix
21	Exp>Cov	N	N	2	Epoxy resin
33	Exp	N	N	2	Chromate, cobalt
24	Exp plus Cov	Y	Y	0	Nil
31	Exp>Cov	N	N	2	Nil
25	Exp plus Cov	Y	Y	5	Chromate, cobalt, nickel, epoxy resin
22	Exp	N	N	4	Chromate, epoxy resin
20	Exp	N	N	3	Chromate, thiuram mix
34	Exp>Cov	y	Y	1	Nil
21	Exp>Cov	N	Y	NT	
19	Exp>Cov	N	N	NT	
27	Exp	N	N	NT	
32	Exp	N	N	NT	
22	Exp>Cov	N	N	NT	
24	Cov	Y	Y	NT	

Exp = exposed areas, Cov = covered areas, NT = not tested, AD = atopic dermatitis

Dermatitis affected exposed areas in 93.75%, covered areas in 62.5%, predominantly exposed areas in 43.8%, both exposed and covered areas with nearly equal severity in 12.5% and solely in covered areas in one case. The most common area involved was forearm.

**Figure 1:**
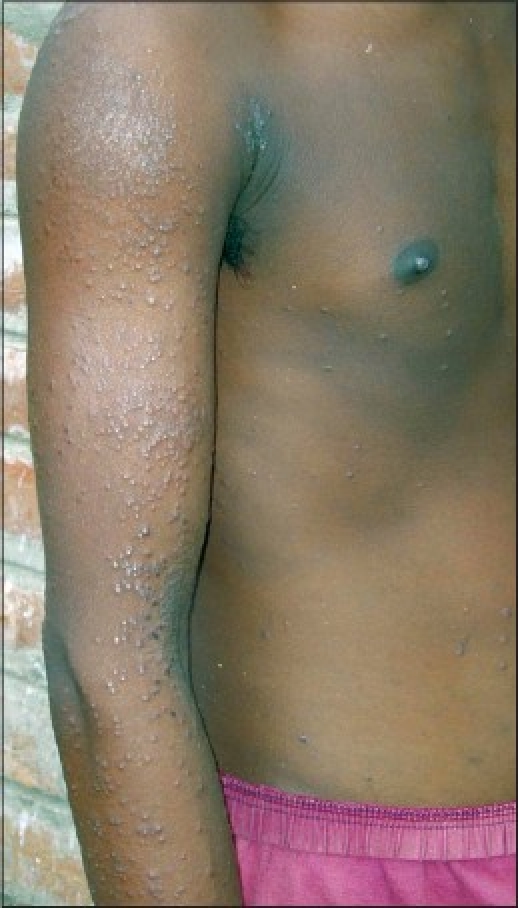
Discrete papular eruptions over exposed areas of forearm and arm with relative sparing trunk

**Figure 2:**
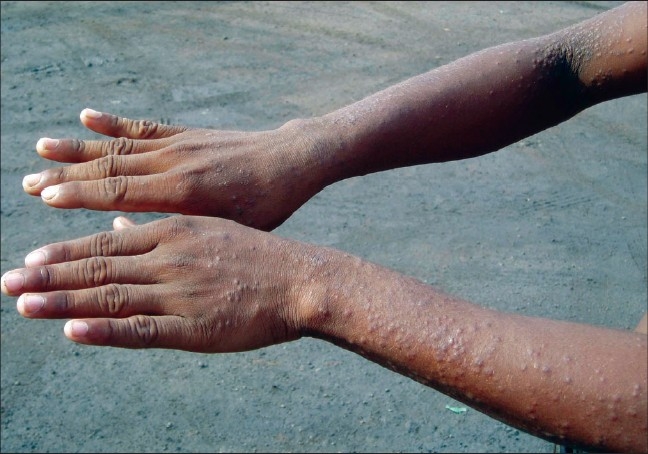
Same patient showing presence of similar papular eruptions over proximal part of dorsum of hand with characteristic sparing of fingers

The eruptions frequently comprised of discrete papules even without other lesions of eczema [Figures 1 and 2].

Atopy was noted in six (37.5%) workers including atopic dermatitis (AD) in four (25%). Pattern of dermatitis among workers with AD who were patch tested (*n* = 4) showed equal prevalence (50%) of irritant contact dermatitis (ICD) and ACD. There was no ICD among nonatopic group (*n* = 6) and all had positive patch test and five (83.33%) had relevant allergies (RAs).

Dermatitis was distributed mostly over the exposed areas like forearm, dorsum of hand and the face and neck [Table 1].

**Figure 3:**
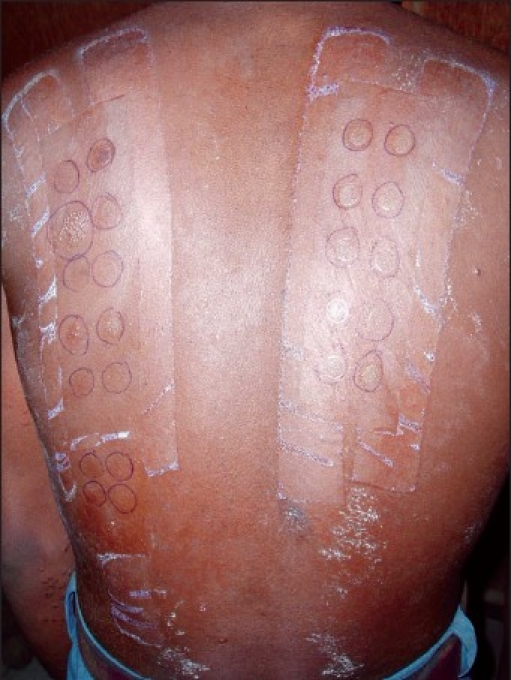
Patch test result after 48 hours showing grade two (++, vesicle) reaction with chromate, grade one (+, papules) reaction with cobalt, nickel and epoxy resin

**Figure 4:**
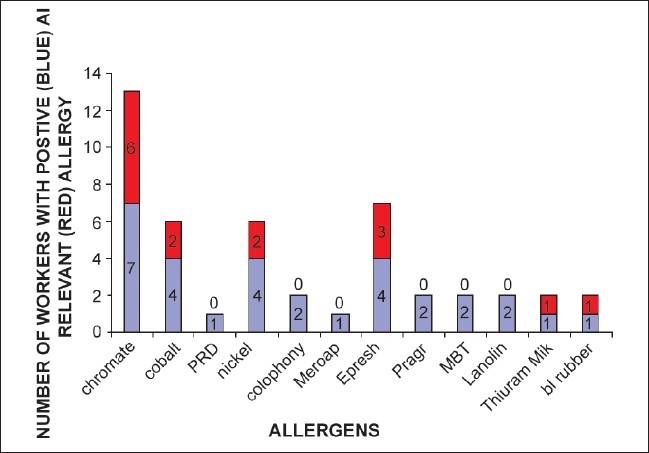
Most frequent allergens with their prevalence in the study population (both positive test and relevant allergy)

Frequently there was multiple positive test in a single patient [Figure 3]. Total 24 positive tests were noted in 8 cases. Relevance was found for 11 positive reactions in 7 cases. The most common relevant allergen was chromate (total positive: 70%, RA: 60% of all tested). Other allergens with RAs were epoxy resin (RA: 30%), cobalt (RA: 20%), nickel (RA: 20%), thiurma mix (RA: 10%), and black rubber mix (RA: 10%) [Figure 4].

**Figure 5:**
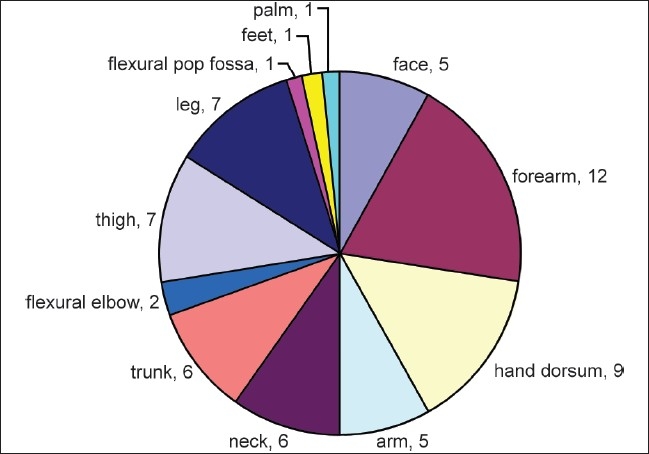
Distribution of disease with number of cases affected

There were 8 irritant reactions noted in 5 workers. Two of them had atopy. Distribution of disease is presented in Figure 5.

## Discussion

Workers engaged in construction work are exposed to various substances. Cement is the most important substance that can cause OCD. Dichromate is the principle allergic component in cement.[8]

Cement also contains nickel and cobalt those are also highly allergic.[5] Epoxy resin is also another important allergen in construction industry.[3] Other substances to which workers may develop OCD are rubber chemical from the protective gloves.[6]

Chromium occurs in different oxidation states from 0-6. Sensitization potential of each form varies widely. Hexavalent chromate (VI) has the highest allergenic potential and has always been an important occupational allergen of clinical significance.[2]

All previous studies from developed countries described chromate allergy among workers of various fields like construction industry, leather and mining industries etc. Prevalence rate of chromate allergy was found to be as high as 19.5% among workers in contrast to 1% among normal population in Switzerland in 1950.[2] The rate among general population in other countries like Denmark,[9] Netherlands,[10] and Italy[11] was as low as 0.5%. Patch testing was done among workers during construction of channel tunnel and a prevalence rate of chromate allergy was found to be 17%.[12]

A review by Bock *et al.,* found the actual prevalence of chromate allergy exclusively among symptomatic workers was even much higher and was in the range that was higher than 45% of workers.[3]

This study was also done only among symptomatic workers and RA to chromate was 60% which was higher than the previous report.

Various steps have been taken in most of the developed countries to reduce the rate of chromate allergy among workers. Addition of iron sulfate in cement has greatly reduced the chance of chromate allergy by reducing the hexavalent chromium to trivalent form which is insoluble and has minimum sensitization and elicitation potential.[1,3,13–15] Extremely high prevalence of chromate allergy in this study among construction workers provided evidence against existence of any such measures in India.

Cobalt and nickel are present in cement and allergy to these metals can occur in the construction workers. Tests have already confirmed the presence these substances in Asian cements.[16] However isolated occupationally relevant allergy to these metals from cement without concomitant allergy to chromate is very rare. It is because cobalt and nickel are present in insoluble form that has very low sensitization potential.[17] Thus, allergy to these metals is usually secondary in the setting of damaged skin incurred upon by the existing chromate allergy. Greater the clinical severity of chromate allergy, more is the chance of sensitization from cobalt and nickel.[17]

Reduction of chromate allergy worldwide has also reduced the prevalence of cobalt and nickel allergy from cement.[18]

In this study also, all the cases with nickel or cobalt allergy had concomitant allergy to chromate.

Epoxy resin has a very high sensitization potential. OCD from this is frequent among workers in construction industry. Clinically it can cause contact allergy even in areas away from hand, like face, due to its volatile nature.[19] The retrospective study from Germany on 33 German and Austrian contact dermatitis units of the Information Network of Departments of Dermatology (IVDK) from 1992-2000 reported a steady overall increase in prevalence of OCD to epoxy resin along with a mild reduction in chromate allergy.[1] ACD to epoxy resin was second most common allergen in that study, being positive in 5.94% of workers. Occupationally RA to epoxy resin in another study was found to be almost 12%. Our study shows even a higher prevalence rate. Positive allergy was noted in 40% and RA in 30% of patch tested cases.

Relevant contact allergy against rubber chemicals was found in 20% of patch tested cases in this study. High prevalence of allergy to thiuram already has been reported in construction workers.[3] Most probably this was from the protective gloves and boots they used.[6]

Hand dermatitis usually predominates among construction workers. Hand dermatitis predominated with involvement of more than 73.7% of the workers in contrast to 11.6% with facial dermatitis and 6.9% with leg dermatitis in Northern Bavaria, from 1990-1999.[3]

In this study, hand dermatitis affecting both palm and dorsum was seen in just one case. Hand dermatitis affecting only dorsal surface was however common and was even second commonest affected area after forearm. The most possible reason why palms were less affected than forearms was the pattern of work done. Concrete blocks were prepared and assembled to make the flyovers. Brick laying was not required unlike other construction works. Mixing of different substances was mostly done by machines and workers had the main job to carry the substances. Moreover, hands had additional protection with gloves. In contrast, exposed parts like forearm, face, and neck had more frequent contact with cement and other substances.

Covered areas like trunk, thigh, or leg were involved in many cases (62.5%). Cases with AD had covered areas affected by aggravation of old dermatitis along with involvement of new areas in the exposed parts. Even non-AD cases also developed dermatitis for the first time in covered areas in 50% of cases. It indicated that allergens regularly came into contact with so-called covered areas also. The results strongly point against the protective capability of garments and also on the frequency of their regular and correct use.

Finally, as forearm was affected most frequently (75%), it warrants urgent implementation of full-sleeved protective garments as an essential requirement for the high-risk workers.

Irritant contact dermatitis was slightly commoner among atopics (ICD:ACD-3:2) and the percentage of cases showing ACD was higher in nonatopic cases. The results contradicted the age-old conception that atopic dermatitis rarely develops ACD.

One of the important observations in this study was the clinical morphology of lesions in many of the cases. These cases presented with itchy discrete papular eruptions without any other signs of eczema [Figures 1 and 2]. We have not come across any previous report that mentions this type of presentation.

Although a large number of studies on this subject was published in the western world, it was difficult to compare the results because the patient selection, study purposes, and methodology used for analysis varied considerably. In contrast to the developed world, there has been no attempt to evaluate the magnitude of this problem, even in small scale in India. This is probably the first Indian study on OCD among construction workers. Although comparisons were made with those studies, it should be remembered that geographical factors and more importantly governmental policies affect strongly the epidemiology of any disease especially the occupational diseases like OCD. Disease patterns in Europe or US may not be directly comparable to those in India due to obvious reasons.

Another important limitation of the present study was the small sample size. Moreover, the overall prevalence of the OCD among workers was not studied. Owing to the small size, the prevalence of OCD to different substances among the workers tested might not reflect the same in construction workers in general. This study indicated the necessity of a comprehensive work on this subject. Finally, we could not perform photopatch test to rule out photocontact dermatitis, although many cases had more severe dermatitis over the exposed areas.

Allergic contact dermititis from the substances used in workplace, like chromate in construction industry leads to lots of wage loss due to absentism. The estimated cost even reached 35 million Euros per year, excluding the costs of sickness days in Germany.[20] Change of profession is nearly impossible in our country because an alternative is rarely available and the very poor technical expertise these workers have can hardly be utilized in other readily available jobs. When this is compounded with the frequency of the problem, it is not difficult to understand the loss in productivity in national level.

Workplace safety guidelines are less strictly supervised in many developing countries, including India. Specific guidelines from the goverment to protect the workers from development of OCD seem to be practically nonexistent in India and many other developing countries.

Quite naturally the private cement manufacturing companies might not be much willing to implement any of the scientific measures to reduce the chromate or epoxy resin allergy in cement in present situation. We hope to see a change in this scenario in the near future.
